# Crystal structure of aqua­[(*E*)-*N*′-(5-bromo-2-oxido­benzyl­idene-κ*O*)benzohydrazidato-κ^2^ *O*,*N*′]dioxidomolybdenum(VI) di­methyl­formamide monosolvate

**DOI:** 10.1107/S2056989015009639

**Published:** 2015-05-28

**Authors:** Radhika Sudheer, M. Sithambaresan, N. R. Sajitha, E. Manoj, M. R. Prathapachandra Kurup

**Affiliations:** a Department of Applied Chemistry, Cochin University of Science and Technology, Kochi 682 022, India; b Department of Chemistry, Faculty of Science, Eastern University, Chenkalady, Sri Lanka; c Dept. of Chemistry, Sree Krishna College, Guruvayur 680 102, Thrissur, Kerala, India

**Keywords:** crystal structure, aroyl hydrazone, supra­molecular, hydrogen bonding, molybdenum complex

## Abstract

The title compound, [Mo(C_14_H_9_BrN_2_O_2_)O_2_(H_2_O)]·C_3_H_7_NO, has a distorted octa­hedral geometry around the Mo atom, with the two terminal oxide groups lying *cis* to each other. The two aromatic rings present in the mol­ecule are almost coplanar, forming a dihedral angle of 1.4 (2)°. The five-membered ring involving the metal atom is puckered, with an amplitude *Q* = 0.358 (2) Å and ϕ = 204.1 (6)°. In the crystal, pairs of inversion-related mol­ecules are linked by O—H⋯N hydrogen bonds. An O—H⋯O hydrogen bond connects the water ligand to the di­methyl­formamide solvent mol­ecule. The crystal packing also features π–π [centroid–centroid distance of 3.688 (2) Å] and C—H⋯O inter­actions.

## Abstract 2

The title compound, [MoO_2_(C_14_H_9_N_2_O_2_Br)H_2_O]·C_3_H_7_NO, has a three-dimensional supra­molecular arrangement *via* a number of inter­molecular C—H⋯O, O—H⋯N and O—H⋯O hydrogen bonds, as well as C—H⋯π and π–π inter­actions.

## Chemical context   

Aroylhydrazones are unique organic compounds characterized by the azomethine group in their mol­ecules (Sheeja *et al.*, 2010 ▸). They exhibit a wide range of applications in the field of biology, optics, catalysis and analytical chemistry. Their broad spectrum of biological activities include anti­microbial (Sreeja *et al.*, 2004 ▸), anti­fungal (Nfor *et al.*, 2013 ▸), anti­viral and anti­neoplastic (Nair *et al.*, 2014 ▸) activities. Biocidal studies reveal that hydrazones can be used as fungicides (Rai, 2006 ▸). Hydrazones are also used as DNA photocleaving agents (Pal *et al.*, 2014 ▸) and even as a reversible photochromic system (Li *et al.*, 2014 ▸). Hydrazone-based mol­ecular switches, metallo­assemblies and sensors have also been developed (Su & Aprahamian, 2014 ▸).
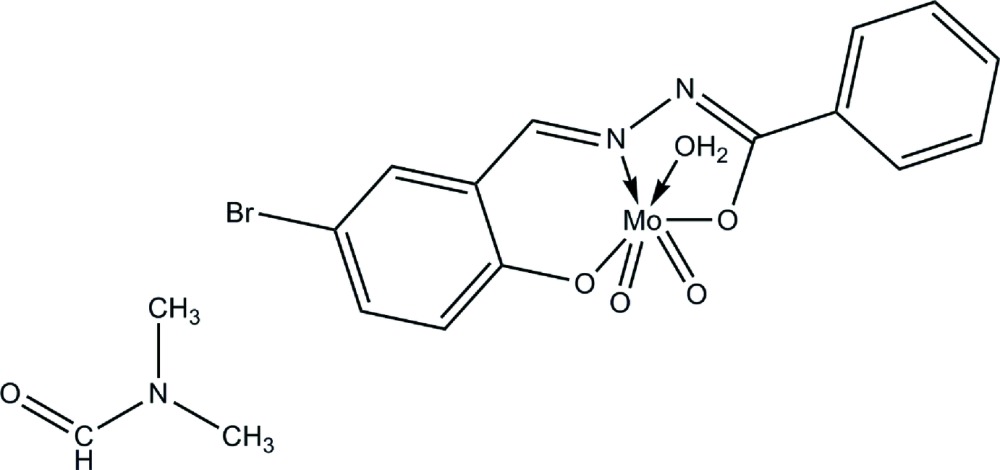


Molybdenum is an important trace metal capable of forming various complexes with versatile organic ligands. Its flexibility in possessing a large number of stable and accessible oxidation states leads to applications in industrial and bio­logical reactions. Molybdenum complexes play a major role in catalytic activity (Maurya *et al.*, 2014 ▸). They are employed as catalysts in olefin epoxidation (Lei & Chelamalla, 2013 ▸), reduction of di­nitro­gen to ammonia (Sengupta *et al.*, 2015 ▸) and oxidation of secondary alcohols (Maurya *et al.*, 2015 ▸). The biological relevance of molybdenum complexes include their application in modelling active sites of molybdoenzymes (Pramanik *et al.*, 2004 ▸) and also their anti­bacterial (Pasayat *et al.*, 2012 ▸), cytotoxic and anti­proliferative activities (Pasayat *et al.*, 2014 ▸).

## Structural commentary   

**Figure 1:**
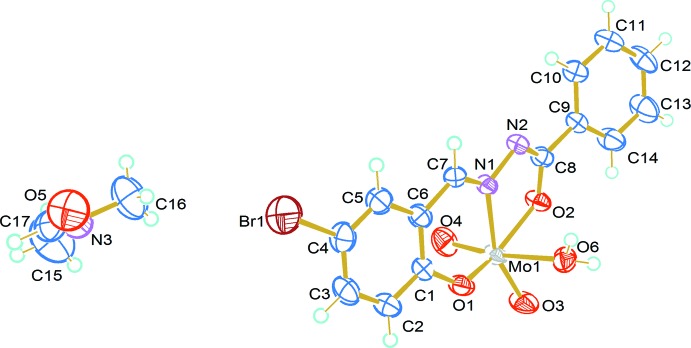
The title compound drawn with 50% probability displacement ellipsoids for the non-H atoms.

The title complex [Mo(C_14_H_9_BrN_2_O_2_)O_2_(H_2_O)]·C_3_H_7_NO crystallizes in the monoclinic space group *P*2_1_/*n*. The complex adopts a distorted octa­hedral geometry around the Mo atom (Fig. 1 ▸) in which the aroylhydrazone coordinates to the metal in a tridentate manner. One di­methyl­formamide solvent mol­ecule is present without any coordination to the metal centre. Two oxygen atoms and one nitro­gen atom of the aroylhydrazone and one of the terminal oxido atoms occupy equatorial positions in the complex. The axial positions are occupied by the other terminal oxygen and the oxygen atom of the water mol­ecule. The two terminal oxido groups are *cis* to each other. The C8—O2 bond length [1.314 (3) Å] is close to the reported C—O single bond length (1.318 Å; Gupta *et al.*, 2007 ▸). The Mo1—O4 and Mo1—O3 bonds of 1.693 (3) and 1.702 (2) Å, respectively, are very close to the reported Mo=O double bond [1.697 (1) Å], indicating that the complex has two Mo=O double bonds (Ebrahimipour *et al.*, 2015 ▸).

The ligand adopts *Z* configurations with respect to the C7—N1 and C8—N2 bonds in the complex, which is clear from C1—C6—C7—N1 and N1—N2—C8—O2 torsion angles [9.8 (5) and −1.4 (4)°, respectively]. This configuration is similar to that of the metal-free ligand (Liu *et al.*, 2006 ▸). The C1–C6 and C9–C14 rings make a dihedral angle of 1.4 (2)° with each other. Ring puckering analysis and least-squares plane calculations show that the Mo1/O1/C1/C6/C7/N1 ring is puckered with puckering amplitude *Q* = 0.358 (2)Å and ϕ = 204.1 (6)°.

## Supra­molecular features   

**Figure 2:**
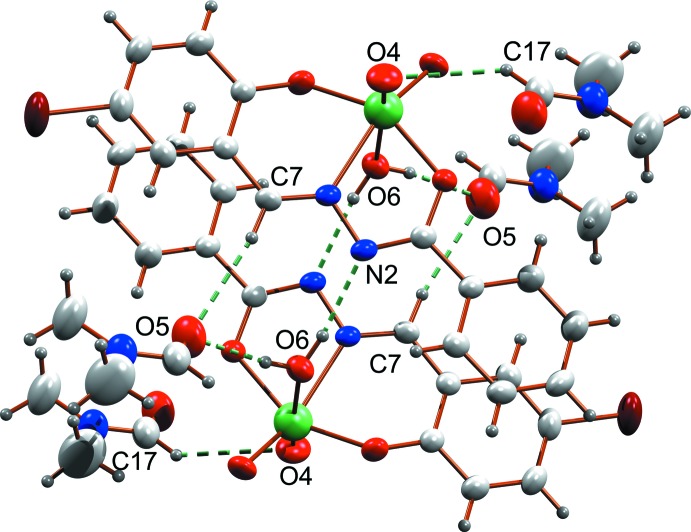
Hydrogen-bonding inter­actions in the title compound.

**Figure 3:**
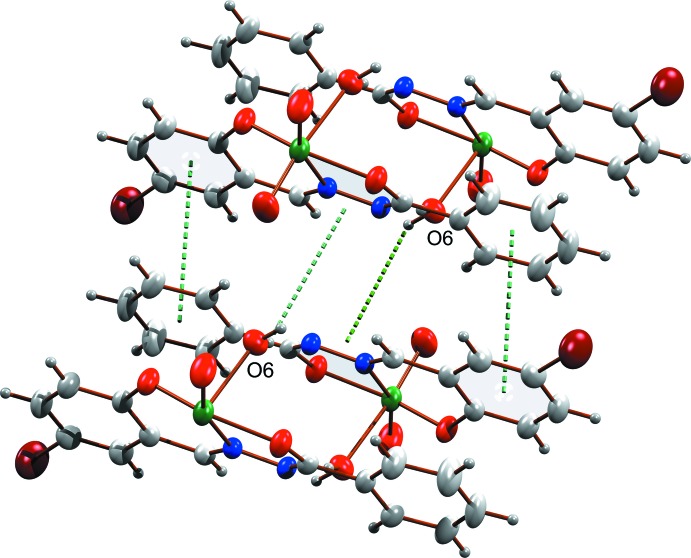
O—H⋯π and π–π inter­actions present in the mol­ecule. Atom O6 is the water O atom.

**Figure 4:**
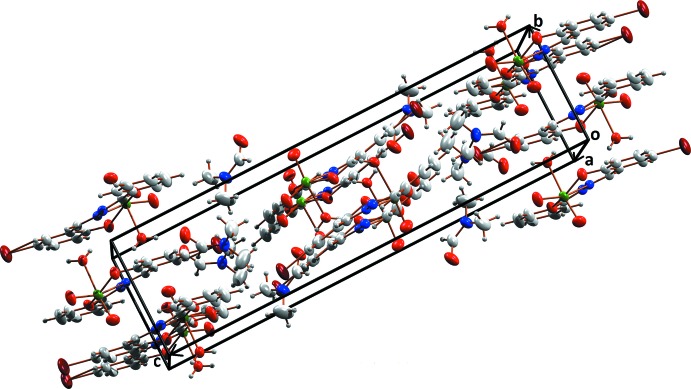
Packing of the mol­ecules, viewed along the *a* axis.

The supra­molecular arrangement of the complex is driven by various types of classical and non-classical hydrogen-bonding inter­actions, in which O4, O5 and N2 act as acceptor atoms (Fig. 2 ▸, Table 1 ▸). There are classical O—H⋯N and O—H⋯O hydrogen-bonding inter­actions with *D*⋯*A* distances 2.891 (4) and 2.701 (4) Å respectively, and a non-classical C—H⋯O inter­action with a *D*⋯*A* distance of 3.421 (5) Å. These inter­actions connect pairs of mol­ecules along with the solvent di­methyl­formamide. The complex mol­ecule is stacked along the *b* axis through two different types of O—H⋯π inter­action (Fig. 3 ▸), with H–centroid distances 2.67 (4) and 2.94 (5) Å and a π–π inter­action between rings C1–C6 and C9–C14(2 − *x*, −*y*, −*z*) with a centroid-centroid distance of 3.688 (2) Å (Fig. 3 ▸). A view of the crystal packing along the *a* axis is given in Fig. 4 ▸.

## Synthesis and crystallization   

The benzoyl hydrazone was synthesized by a reported procedure (Liu *et al.*, 2006 ▸). A methano­lic solution of benzhydrazide (0.0680 g, 0.5 mmol) was refluxed with a methano­lic solution of 5-bromo­salicyl­aldehyde (0.1005 g, 0.5 mmol) continuously for 3 h. The reaction mixture was kept aside for slow evaporation at room temperature. After 2–3 days, a pale-yellow compound formed, and was washed with methanol and dried under vacuum.

The complex was synthesized by refluxing a methano­lic solution of benzoyl hydrazone (0.1595 g, 0.5 mmol) and MoCl_5_ (0.1362 g, 0.5 mmol) for 3 h. The brown precipitate obtained was filtered, washed with methanol, dried and recrystallized from di­methyl­formamide (yield, 0.1688g, 63%). FT–IR (KBr, cm^−1^) ν_max_: 3400, 3194, 1657, 1546, 1345, 937, 810.

## Refinement   

Crystal data, data collection and structure refinement details are summarized in Table 2 ▸. All C-bound H atoms were placed in calculated positions, guided by difference Fourier maps, with C—H bond lengths of 0.93–0.96 Å and with *U*_iso_(H) = 1.2*U*_eq_(carrier) or 1.5*U*_eq_(methyl C). The O—H distances were restrained with 1,2 and 1,3 distance restraints of 0.86 (1) and 1.36 (2) Å. Reflections (0 0 2), (1 0 1) and (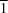 0 1), which were obscured by the beam stop, were omitted.

## Supplementary Material

Crystal structure: contains datablock(s) I. DOI: 10.1107/S2056989015009639/pk2550sup1.cif

Structure factors: contains datablock(s) I. DOI: 10.1107/S2056989015009639/pk2550Isup2.hkl

CCDC reference: 1401828

Additional supporting information:  crystallographic information; 3D view; checkCIF report

## Figures and Tables

**Table 1:** Hydrogen-bond geometry (, )

*D*H*A*	*D*H	H*A*	*D* *A*	*D*H*A*
C7H7O5^i^	0.93	2.51	3.421(5)	168
C17H17O4^ii^	0.93	2.63	3.404(5)	141
O6H6*A*N2^iii^	0.86(1)	2.04(1)	2.891(3)	173(3)
O6H6*B*O5^iv^	0.86(1)	1.85(1)	2.701(4)	171(4)

Symmetry codes: (i) 
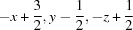
; (ii) 
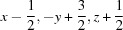
; (iii) 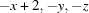; (iv) 
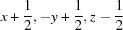
.

**Table 2:** Experimental details

Crystal data	
Chemical formula	[Mo(C_14_H_9_BrN_2_O_2_)O_2_(H_2_O)]C_3_H_7_NO
*M*_r_	536.19
Crystal system, space group	Monoclinic, *P*2_1_/*n*
Temperature (K)	296
*a*, *b*, *c* ()	10.8581(8), 7.1145(5), 25.998(2)
()	93.900(3)
*V* (^3^)	2003.7(3)
*Z*	4
Radiation type	Mo *K*
(mm^1^)	2.69
Crystal size (mm)	0.40 0.15 0.10

Data collection	
Diffractometer	Bruker APEXII CCD
Absorption correction	Multi-scan (*SADABS*; Bruker, 2004 ▸)
*T*_min_, *T*_max_	0.355, 0.447
No. of measured, independent and observed [*I* > 2(*I*)] reflections	14880, 4957, 3710
*R*_int_	0.027
(sin /)_max_ (^1^)	0.667

Refinement	
*R*[*F* ^2^ > 2(*F* ^2^)], *wR*(*F* ^2^), *S*	0.043, 0.096, 1.08
No. of reflections	4957
No. of parameters	264
No. of restraints	3
H-atom treatment	H atoms treated by a mixture of independent and constrained refinement
_max_, _min_ (e ^3^)	1.31, 0.89

Computer programs: *APEX2*, *SAINT* and *XPREP* (Bruker, 2004 ▸), *SHELXS2014* and *SHELXL97* (Sheldrick, 2008 ▸), *SHELXL2014* (Sheldrick, 2015 ▸), *ORTEP-3* (Burnett Johnson, 1996 ▸), *DIAMOND* (Brandenburg, 2010 ▸) and *publCIF* (Westrip, 2010 ▸).

## supplementary crystallographic information

### Crystal data

**Table:**

[Mo(C_14_H_9_BrN_2_O_2_)O_2_(H_2_O)]·C_3_H_7_NO	*F*(000) = 1064
*M**_r_* = 536.19	*D*_x_ = 1.777 Mg m^−^^3^
Monoclinic, *P*2_1_/*n*	Mo *K*α radiation, λ = 0.71073 Å
*a* = 10.8581 (8) Å	Cell parameters from 5189 reflections
*b* = 7.1145 (5) Å	θ = 2.9–28.1°
*c* = 25.998 (2) Å	µ = 2.69 mm^−^^1^
β = 93.900 (3)°	*T* = 296 K
*V* = 2003.7 (3) Å^3^	Needle, yellow
*Z* = 4	0.40 × 0.15 × 0.10 mm

### Data collection

**Table:**

Bruker APEXII CCD diffractometer	3710 reflections with *I* > 2σ(*I*)
Radiation source: fine-focus sealed tube	*R*_int_ = 0.027
ω and φ scan	θ_max_ = 28.3°, θ_min_ = 2.9°
Absorption correction: multi-scan (*SADABS*; Bruker, 2004)	*h* = −14→14
*T*_min_ = 0.355, *T*_max_ = 0.447	*k* = −8→9
14880 measured reflections	*l* = −34→31
4957 independent reflections	

### Refinement

**Table:**

Refinement on *F*^2^	Hydrogen site location: mixed
Least-squares matrix: full	H atoms treated by a mixture of independent and constrained refinement
*R*[*F*^2^ > 2σ(*F*^2^)] = 0.043	*w* = 1/[σ^2^(*F*_o_^2^) + (0.0418*P*)^2^ + 1.3003*P*] where *P* = (*F*_o_^2^ + 2*F*_c_^2^)/3
*wR*(*F*^2^) = 0.096	(Δ/σ)_max_ = 0.001
*S* = 1.08	Δρ_max_ = 1.31 e Å^−^^3^
4957 reflections	Δρ_min_ = −0.88 e Å^−^^3^
264 parameters	Extinction correction: *SHELXL2014* (Sheldrick, 2015), Fc^*^=kFc[1+0.001xFc^2^λ^3^/sin(2θ)]^-1/4^
3 restraints	Extinction coefficient: 0.0007 (2)

### Special details

**Table:**

Geometry. All e.s.d.'s (except the e.s.d. in the dihedral angle between two l.s. planes) are estimated using the full covariance matrix. The cell e.s.d.'s are taken into account individually in the estimation of e.s.d.'s in distances, angles and torsion angles; correlations between e.s.d.'s in cell parameters are only used when they are defined by crystal symmetry. An approximate (isotropic) treatment of cell e.s.d.'s is used for estimating e.s.d.'s involving l.s. planes.

### Fractional atomic coordinates and isotropic or equivalent isotropic displacement parameters (Å^2^)

**Table:**

	*x*	*y*	*z*	*U*_iso_*/*U*_eq_	
C1	0.6397 (3)	0.2038 (4)	0.03480 (13)	0.0315 (7)	
C2	0.5256 (3)	0.2057 (5)	0.05560 (14)	0.0399 (8)	
H2	0.4549	0.1793	0.0346	0.048*	
C3	0.5153 (3)	0.2461 (5)	0.10676 (14)	0.0453 (9)	
H3	0.4382	0.2497	0.1203	0.054*	
C4	0.6213 (3)	0.2812 (5)	0.13790 (14)	0.0433 (8)	
C5	0.7351 (3)	0.2765 (5)	0.11877 (13)	0.0402 (8)	
H5	0.8051	0.2982	0.1406	0.048*	
C6	0.7471 (3)	0.2391 (4)	0.06655 (12)	0.0312 (6)	
C7	0.8694 (3)	0.2411 (4)	0.04814 (12)	0.0317 (6)	
H7	0.9367	0.2462	0.0722	0.038*	
C8	1.0185 (3)	0.2494 (4)	−0.06177 (12)	0.0299 (6)	
C9	1.1389 (3)	0.2485 (4)	−0.08474 (12)	0.0315 (6)	
C10	1.2469 (3)	0.2848 (5)	−0.05437 (13)	0.0354 (7)	
H10	1.2436	0.3138	−0.0196	0.042*	
C11	1.3591 (3)	0.2772 (5)	−0.07643 (15)	0.0443 (9)	
H11	1.4315	0.3026	−0.0564	0.053*	
C12	1.3649 (3)	0.2328 (6)	−0.12723 (16)	0.0524 (10)	
H12	1.4410	0.2266	−0.1415	0.063*	
C13	1.2584 (4)	0.1972 (7)	−0.15748 (16)	0.0620 (12)	
H13	1.2624	0.1666	−0.1921	0.074*	
C14	1.1456 (3)	0.2070 (6)	−0.13613 (14)	0.0498 (10)	
H14	1.0735	0.1853	−0.1567	0.060*	
C15	0.3158 (5)	1.0771 (9)	0.2643 (2)	0.105 (2)	
H15A	0.2832	1.0438	0.2303	0.158*	
H15B	0.3700	1.1831	0.2624	0.158*	
H15C	0.2491	1.1090	0.2852	0.158*	
C16	0.4848 (5)	0.8476 (8)	0.2605 (2)	0.0858 (16)	
H16A	0.5059	0.7242	0.2732	0.129*	
H16B	0.5547	0.9293	0.2661	0.129*	
H16C	0.4614	0.8408	0.2243	0.129*	
C17	0.3545 (4)	0.8450 (7)	0.33094 (16)	0.0553 (10)	
H17	0.2877	0.8967	0.3465	0.066*	
N1	0.8897 (2)	0.2360 (3)	0.00006 (10)	0.0284 (5)	
N2	1.0134 (2)	0.2354 (4)	−0.01231 (10)	0.0308 (6)	
N3	0.3835 (3)	0.9198 (5)	0.28720 (12)	0.0550 (8)	
O1	0.64511 (18)	0.1587 (4)	−0.01536 (9)	0.0394 (5)	
O2	0.92037 (18)	0.2583 (3)	−0.09418 (9)	0.0367 (5)	
O3	0.6772 (2)	0.1701 (4)	−0.12470 (9)	0.0512 (7)	
O4	0.7253 (2)	0.4738 (4)	−0.06416 (11)	0.0539 (7)	
O5	0.4074 (3)	0.7140 (5)	0.35275 (12)	0.0743 (9)	
O6	0.8105 (2)	−0.0693 (3)	−0.06282 (10)	0.0384 (5)	
Br1	0.60802 (5)	0.33679 (9)	0.20859 (2)	0.07719 (18)	
Mo1	0.75010 (2)	0.23944 (4)	−0.06785 (2)	0.03247 (10)	
H6A	0.858 (3)	−0.117 (5)	−0.0387 (9)	0.046 (11)*	
H6B	0.834 (4)	−0.118 (6)	−0.0907 (8)	0.092 (18)*	

### Atomic displacement parameters (Å^2^)

**Table:**

	*U*^11^	*U*^22^	*U*^33^	*U*^12^	*U*^13^	*U*^23^
C1	0.0296 (14)	0.0328 (18)	0.0324 (16)	0.0022 (12)	0.0037 (12)	0.0019 (13)
C2	0.0290 (15)	0.047 (2)	0.044 (2)	−0.0013 (13)	0.0035 (14)	0.0032 (16)
C3	0.0371 (16)	0.057 (2)	0.043 (2)	0.0015 (17)	0.0154 (15)	0.0038 (18)
C4	0.052 (2)	0.047 (2)	0.0325 (18)	−0.0001 (16)	0.0122 (15)	0.0028 (16)
C5	0.0396 (17)	0.048 (2)	0.0326 (17)	0.0010 (15)	0.0016 (13)	0.0010 (16)
C6	0.0303 (14)	0.0311 (16)	0.0322 (16)	0.0032 (13)	0.0022 (11)	0.0040 (14)
C7	0.0280 (13)	0.0354 (17)	0.0309 (16)	0.0003 (13)	−0.0030 (11)	−0.0033 (15)
C8	0.0270 (13)	0.0281 (16)	0.0346 (16)	−0.0016 (13)	0.0017 (11)	0.0037 (14)
C9	0.0262 (13)	0.0333 (17)	0.0352 (17)	0.0019 (13)	0.0033 (12)	0.0053 (15)
C10	0.0318 (15)	0.038 (2)	0.0363 (18)	−0.0015 (13)	0.0013 (13)	0.0070 (14)
C11	0.0276 (14)	0.056 (2)	0.049 (2)	−0.0003 (15)	0.0004 (14)	0.0156 (18)
C12	0.0327 (16)	0.074 (3)	0.052 (2)	0.0104 (18)	0.0146 (16)	0.014 (2)
C13	0.047 (2)	0.101 (4)	0.039 (2)	0.009 (2)	0.0117 (17)	0.000 (2)
C14	0.0346 (17)	0.077 (3)	0.038 (2)	−0.0012 (17)	0.0021 (15)	−0.0014 (19)
C15	0.102 (4)	0.099 (5)	0.116 (5)	0.026 (4)	0.017 (4)	0.044 (4)
C16	0.083 (4)	0.103 (4)	0.075 (4)	0.012 (3)	0.035 (3)	0.021 (3)
C17	0.056 (2)	0.069 (3)	0.042 (2)	−0.008 (2)	0.0081 (18)	−0.005 (2)
N1	0.0229 (11)	0.0293 (14)	0.0326 (14)	0.0023 (10)	0.0002 (9)	−0.0006 (12)
N2	0.0217 (11)	0.0353 (15)	0.0352 (14)	0.0005 (10)	0.0007 (10)	−0.0010 (12)
N3	0.060 (2)	0.062 (2)	0.0432 (19)	0.0035 (16)	0.0052 (15)	0.0134 (16)
O1	0.0249 (10)	0.0582 (15)	0.0347 (13)	−0.0035 (10)	0.0002 (9)	−0.0062 (12)
O2	0.0259 (9)	0.0533 (15)	0.0307 (11)	0.0017 (10)	−0.0001 (8)	0.0064 (11)
O3	0.0334 (12)	0.086 (2)	0.0321 (13)	0.0029 (12)	−0.0099 (10)	−0.0021 (13)
O4	0.0500 (15)	0.0474 (16)	0.0641 (18)	0.0155 (12)	0.0026 (13)	0.0089 (13)
O5	0.087 (2)	0.088 (3)	0.0481 (18)	−0.0023 (18)	0.0050 (16)	0.0220 (17)
O6	0.0417 (13)	0.0406 (14)	0.0323 (13)	0.0056 (10)	−0.0025 (10)	−0.0038 (12)
Br1	0.0820 (3)	0.1149 (5)	0.0373 (2)	−0.0145 (3)	0.0234 (2)	−0.0067 (3)
Mo1	0.02312 (13)	0.04399 (19)	0.02970 (15)	0.00441 (12)	−0.00257 (9)	0.00338 (13)

### Geometric parameters (Å, º)

**Table:**

C1—O1	1.348 (4)	C12—H12	0.9300
C1—C2	1.386 (4)	C13—C14	1.380 (5)
C1—C6	1.405 (4)	C13—H13	0.9300
C2—C3	1.373 (5)	C14—H14	0.9300
C2—H2	0.9300	C15—N3	1.445 (6)
C3—C4	1.384 (5)	C15—H15A	0.9600
C3—H3	0.9300	C15—H15B	0.9600
C4—C5	1.364 (5)	C15—H15C	0.9600
C4—Br1	1.895 (4)	C16—N3	1.435 (5)
C5—C6	1.398 (4)	C16—H16A	0.9600
C5—H5	0.9300	C16—H16B	0.9600
C6—C7	1.441 (4)	C16—H16C	0.9600
C7—N1	1.284 (4)	C17—O5	1.215 (5)
C7—H7	0.9300	C17—N3	1.313 (5)
C8—N2	1.295 (4)	C17—H17	0.9300
C8—O2	1.314 (3)	N1—N2	1.403 (3)
C8—C9	1.474 (4)	N1—Mo1	2.247 (2)
C9—C14	1.375 (5)	O1—Mo1	1.924 (2)
C9—C10	1.393 (4)	O2—Mo1	2.019 (2)
C10—C11	1.382 (4)	O3—Mo1	1.702 (2)
C10—H10	0.9300	O4—Mo1	1.693 (3)
C11—C12	1.363 (5)	O6—Mo1	2.293 (2)
C11—H11	0.9300	O6—H6A	0.857 (10)
C12—C13	1.377 (6)	O6—H6B	0.856 (10)
			
O1—C1—C2	118.6 (3)	N3—C15—H15A	109.5
O1—C1—C6	121.4 (3)	N3—C15—H15B	109.5
C2—C1—C6	119.9 (3)	H15A—C15—H15B	109.5
C3—C2—C1	120.9 (3)	N3—C15—H15C	109.5
C3—C2—H2	119.5	H15A—C15—H15C	109.5
C1—C2—H2	119.5	H15B—C15—H15C	109.5
C2—C3—C4	119.0 (3)	N3—C16—H16A	109.5
C2—C3—H3	120.5	N3—C16—H16B	109.5
C4—C3—H3	120.5	H16A—C16—H16B	109.5
C5—C4—C3	121.4 (3)	N3—C16—H16C	109.5
C5—C4—Br1	119.3 (3)	H16A—C16—H16C	109.5
C3—C4—Br1	119.3 (3)	H16B—C16—H16C	109.5
C4—C5—C6	120.4 (3)	O5—C17—N3	125.6 (4)
C4—C5—H5	119.8	O5—C17—H17	117.2
C6—C5—H5	119.8	N3—C17—H17	117.2
C5—C6—C1	118.4 (3)	C7—N1—N2	117.0 (2)
C5—C6—C7	118.0 (3)	C7—N1—Mo1	127.82 (19)
C1—C6—C7	123.6 (3)	N2—N1—Mo1	115.16 (18)
N1—C7—C6	123.1 (3)	C8—N2—N1	109.5 (2)
N1—C7—H7	118.5	C17—N3—C16	120.7 (4)
C6—C7—H7	118.5	C17—N3—C15	121.7 (4)
N2—C8—O2	123.6 (3)	C16—N3—C15	117.6 (4)
N2—C8—C9	120.0 (3)	C1—O1—Mo1	132.99 (19)
O2—C8—C9	116.3 (3)	C8—O2—Mo1	120.02 (19)
C14—C9—C10	119.5 (3)	Mo1—O6—H6A	126 (2)
C14—C9—C8	120.1 (3)	Mo1—O6—H6B	116 (3)
C10—C9—C8	120.4 (3)	H6A—O6—H6B	105 (2)
C11—C10—C9	119.3 (3)	O4—Mo1—O3	105.57 (13)
C11—C10—H10	120.3	O4—Mo1—O1	98.63 (11)
C9—C10—H10	120.3	O3—Mo1—O1	105.44 (11)
C12—C11—C10	120.7 (3)	O4—Mo1—O2	96.11 (11)
C12—C11—H11	119.7	O3—Mo1—O2	96.16 (10)
C10—C11—H11	119.7	O1—Mo1—O2	149.37 (9)
C11—C12—C13	120.3 (3)	O4—Mo1—N1	93.86 (11)
C11—C12—H12	119.9	O3—Mo1—N1	158.18 (11)
C13—C12—H12	119.9	O1—Mo1—N1	80.79 (9)
C12—C13—C14	119.6 (4)	O2—Mo1—N1	71.52 (9)
C12—C13—H13	120.2	O4—Mo1—O6	170.43 (11)
C14—C13—H13	120.2	O3—Mo1—O6	83.47 (11)
C9—C14—C13	120.6 (3)	O1—Mo1—O6	81.64 (10)
C9—C14—H14	119.7	O2—Mo1—O6	79.51 (9)
C13—C14—H14	119.7	N1—Mo1—O6	76.70 (9)
			
O1—C1—C2—C3	178.4 (3)	C8—C9—C10—C11	177.9 (3)
C6—C1—C2—C3	1.5 (5)	C9—C10—C11—C12	−0.7 (5)
C1—C2—C3—C4	−1.4 (5)	C10—C11—C12—C13	0.8 (6)
C2—C3—C4—C5	0.0 (6)	C11—C12—C13—C14	0.2 (7)
C2—C3—C4—Br1	−179.9 (3)	C10—C9—C14—C13	1.3 (6)
C3—C4—C5—C6	1.2 (5)	C8—C9—C14—C13	−177.0 (4)
Br1—C4—C5—C6	−178.8 (3)	C12—C13—C14—C9	−1.2 (7)
C4—C5—C6—C1	−1.0 (5)	C6—C7—N1—N2	−178.9 (3)
C4—C5—C6—C7	178.3 (3)	C6—C7—N1—Mo1	3.7 (4)
O1—C1—C6—C5	−177.1 (3)	O2—C8—N2—N1	−1.4 (4)
C2—C1—C6—C5	−0.3 (5)	C9—C8—N2—N1	−178.9 (3)
O1—C1—C6—C7	3.6 (5)	C7—N1—N2—C8	−173.6 (3)
C2—C1—C6—C7	−179.6 (3)	Mo1—N1—N2—C8	4.1 (3)
C5—C6—C7—N1	−169.5 (3)	O5—C17—N3—C16	0.4 (7)
C1—C6—C7—N1	9.8 (5)	O5—C17—N3—C15	−179.4 (5)
N2—C8—C9—C14	160.5 (3)	C2—C1—O1—Mo1	146.6 (3)
O2—C8—C9—C14	−17.2 (5)	C6—C1—O1—Mo1	−36.6 (4)
N2—C8—C9—C10	−17.8 (5)	N2—C8—O2—Mo1	−2.5 (4)
O2—C8—C9—C10	164.5 (3)	C9—C8—O2—Mo1	175.1 (2)
C14—C9—C10—C11	−0.4 (5)		

### Hydrogen-bond geometry (Å, º)

**Table:**

*D*—H···*A*	*D*—H	H···*A*	*D*···*A*	*D*—H···*A*
C7—H7···O5^i^	0.93	2.51	3.421 (5)	168
C17—H17···O4^ii^	0.93	2.63	3.404 (5)	141
O6—H6*A*···N2^iii^	0.86 (1)	2.04 (1)	2.891 (3)	173 (3)
O6—H6*B*···O5^iv^	0.86 (1)	1.85 (1)	2.701 (4)	171 (4)

Symmetry codes: (i) −*x*+3/2, *y*−1/2, −*z*+1/2; (ii) *x*−1/2, −*y*+3/2, *z*+1/2; (iii) −*x*+2, −*y*, −*z*; (iv) *x*+1/2, −*y*+1/2, *z*−1/2.
